# Review of imaging techniques for evaluating morphological and functional responses to the treatment of bone metastases in prostate and breast cancer

**DOI:** 10.1007/s12094-022-02784-0

**Published:** 2022-02-13

**Authors:** J. Orcajo-Rincon, J. Muñoz-Langa, J. M. Sepúlveda-Sánchez, G. C. Fernández-Pérez, M. Martínez, E. Noriega-Álvarez, S. Sanz-Viedma, J. C. Vilanova, A. Luna

**Affiliations:** 1 Nuclear Medicine Department, Hospital Universitario Gregorio Marañón, Madrid, Spain; 2 Medical Oncology, Hospital Dr. Arnau de Vilanova, Valencia, Spain; 3 Medical Oncology, Hospital Universitario, 12 de Octubre, Madrid, Spain; 4 Radiology, Hospital Universitario Río Hortega, Valladolid, Spain; 5 Medical Oncology, Hospital del Mar, Barcelona, Spain; 6 Nuclear Medicine, General University Hospital of Ciudad Real, Ciudad Real, Spain; 7 Nuclear Medicine, Hospital Universitario Virgen de la Victoria, Instituto de Investigación Biomédica de Málaga (IBIMA), Málaga, Spain; 8 Radiology, Clínica Girona, Institute Diagnostic Imaging (IDI), University of Girona, Girona, Spain; 9 Radiology, HT médica, Clínica Las Nieves, Calle Carmelo Torres, 2, 23007 Jaén, Spain

**Keywords:** Bone metastases, Prostate cancer, Breast cancer, Response, Consensus

## Abstract

Bone metastases are very common complications associated with certain types of cancers that frequently negatively impact the quality of life and functional status of patients; thus, early detection is necessary for the implementation of immediate therapeutic measures to reduce the risk of skeletal complications and improve survival and quality of life. There is no consensus or universal standard approach for the detection of bone metastases in cancer patients based on imaging. Endorsed by the Spanish Society of Medical Oncology (SEOM), the Spanish Society of Medical Radiology (SERAM), and the Spanish Society of Nuclear Medicine and Molecular Imaging (SEMNIM) a group of experts met to discuss and provide an up-to-date review of our current understanding of the biological mechanisms through which tumors spread to the bone and describe the imaging methods available to diagnose bone metastasis and monitor their response to oncological treatment, focusing on patients with breast and prostate cancer. According to current available data, the use of next-generation imaging techniques, including whole-body diffusion-weighted MRI, PET/CT, and PET/MRI with novel radiopharmaceuticals, is recommended instead of the classical combination of CT and bone scan in detection, staging and response assessment of bone metastases from prostate and breast cancer.

**Clinical trial registration:** Not applicable.

## Introduction

**Fig. 1:**
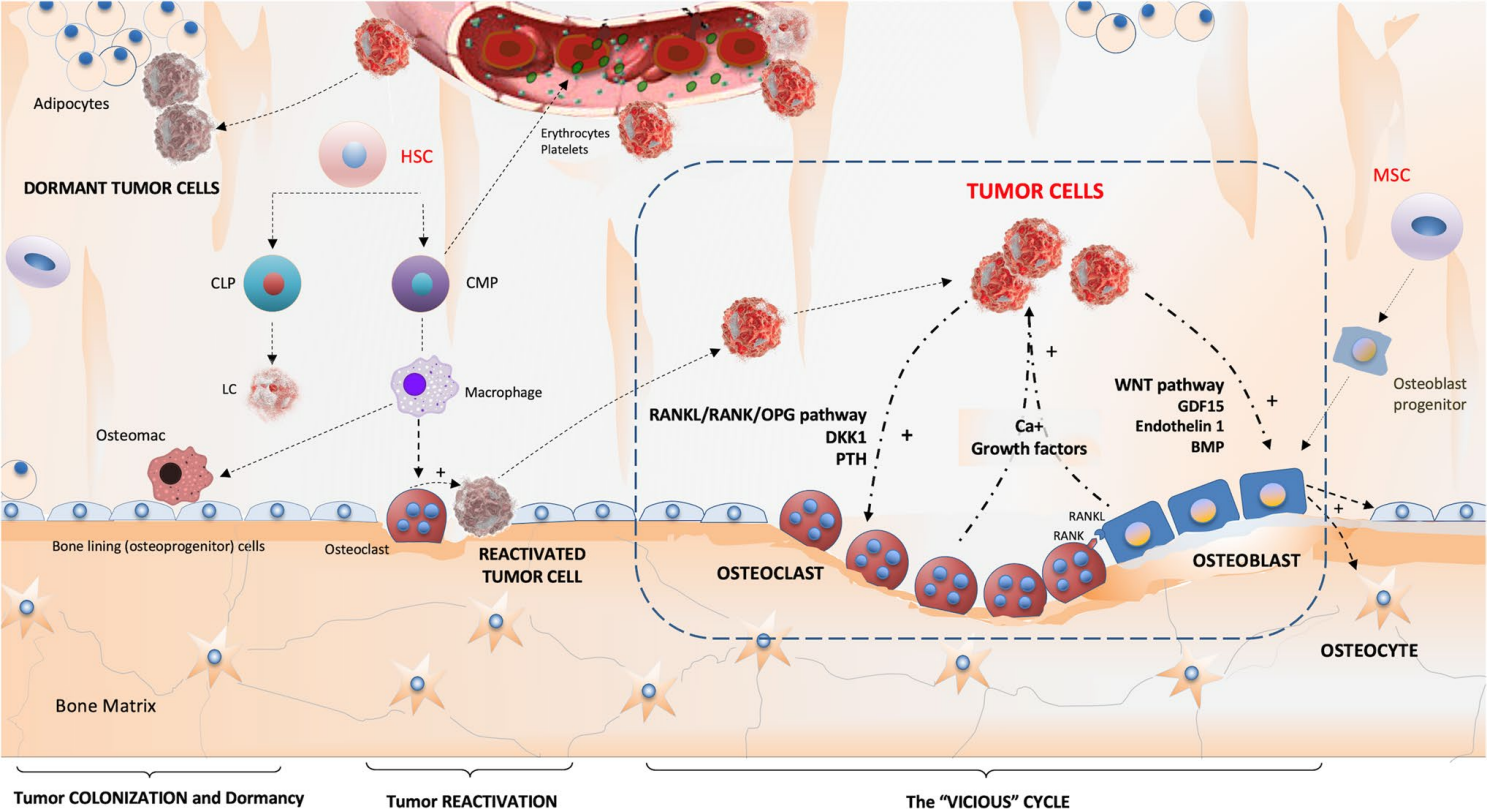
Drawing representing the physiopathology of bone metastases. The bone marrow (BM) is an attractive niche for certain tumor cells, owing to a number of physical, biochemical, and cellular properties. The relationship between the bone marrow niche and infiltrating tumor cells is dynamic. Tumor cells colonize, alter and hijack the niche, making the microenvironment even more hospitable for them and interacting with osteoblasts and osteoclasts causing osteoblastic and osteolytic lesions, which really represent a continuum, and facilitating tumor growth (the so-called “Vicious Cycle”). *BMP* bone morphogenic proteins, *CLP* common lymphoid precursor, *CMP* common myeloid precursor, *DKK-1* Dickkopf1, *GDF15* growth differentiation factor 15, *LC* lymphoid cell, *MSC* mesenchymal stromal cell, *MHSC* multipotential haematopoietic stem cell, *osteomac* osteal macrophage, *OPG* osteoprotegerin, *PTH* parathormone, *RANKL* receptor activator for nuclear factor κB ligand. Courtesy of Roberto García Figueiras, Radiology department, Complexo Hospitalario Santiago de Compostela (Spain)

Bone metastases are very common complications in certain types of cancer; their physiopathology is shown in Fig. 1. In necropsy studies, bone metastases have been reported in up to 70% of patients with prostate or breast cancer; in the latter case, bone is the first metastatic site in approximately 50% of patients [1]. The first metastatic site for bone metastases is the axial skeleton in 60–70% of patients with prostate cancer [2, 3] and in 40–50% of patients with breast cancer [3, 4]. Bone dissemination is also important because it causes significant comorbidities. Accordingly, the most frequent skeletal-related events (SREs) are spinal cord/nerve root compression (3.1% of patients with prostate cancer and bone metastasis; 2% of patients with breast cancer and bone metastases), pathological fractures (18% of patients with prostate cancer and bone metastasis and 12% of patients with breast cancer and bone metastases), and bone marrow infiltration (2% in patients with prostate cancer and 4% in those with breast cancer) [5, 6]. These complications negatively impact the patients’ quality of life and functional status; thus, early detection is necessary for the implementation of immediate therapeutic measures to reduce the risk of skeletal complications and improve survival and quality of life [7].

Early detection of bone metastases is also essential for accurate tumor staging and optimal oncological treatment. In breast cancer, there is little evidence that early detection of bone metastases in asymptomatic or minimally symptomatic patients has a significant impact on quality of life and survival [8]. However, as current therapies for advanced metastatic cancer improve, the early treatment of small-volume metastases may become a more effective strategy. In fact, in metastatic prostate cancer, it has been shown that active treatment of asymptomatic or minimally symptomatic disease leads to benefits in disease-free survival and overall survival [9, 10]; thus, some clinical guidelines and consensus statements now recommend the prospective detection of metastatic disease in patients with asymptomatic prostate cancer, aiming at earlier active treatment either with chemotherapy or androgen deprivation therapy [11, 12]. Furthermore, the therapeutic strategy for bone metastases of prostate cancer changes according to the number of metastases and their location [13]. In oligometastatic breast cancer, a prospective phase II multicenter trial found that radical radiotherapy to all metastatic sites may improve progression-free survival [14]. Similar improvement in PFS was found in a randomized phase II trial where 99 patients with several tumor types, including breast (*n* = 18) and prostate (*n* = 16) cancer, and 1–5 metastatic lesions were randomized to receive palliative standard-of-care (SOC) or SOC plus stereotactic radiotherapy; in this trial an improvement in the 5-year OS rate from 17% among those who received SOC to 42% among those assigned to SOC plus stereotactic radiotherapy [15].

At staging, the objectives of imaging techniques are to identify the exact number of metastatic foci and their location, to quantify the tumor load and to exclude complications, such as pathological fractures or spinal cord compression. In addition, imaging can guide biopsies if considered necessary and has a growing role in therapy monitoring [16].

There are two pure radiographic manifestations of bone metastases: osteoblastic lesions, when bone-forming processes prevail and osteolytic lesions when resorptive processes are dominant; however, it is common to find mixed features in radiological assessment. Osteolytic metastases cause more clinical complications [5].

There is no consensus or universal standard approach for detecting bone metastases in cancer patients with imaging. The choice of the most appropriate imaging strategy should be selected according to the clinical presentation and the underlying histological tumor type because osteoblastic and osteolytic patterns differ. In general, metastatic bone X-ray series are not considered a useful tool in the assessment of bone metastases [17]. Bone scintigraphy is commonly performed in the initial staging of patients with known cancer capable of producing mixed metastases (lytic and blastic) or purely blastic metastases, such as prostate cancer. However, the sensitivity and accuracy of bone scintigraphy in bone metastasis detection is significantly inferior to those of more recent modalities, such as whole-body magnetic resonance imaging (WB-MRI) with diffusion-weighted imaging (DWI) [18] or fluorodeoxyglucose-positron emission tomography/computed tomography (FDG-PET/CT) [19, 20]. These recent technologies are also known as next-generation imaging (NGI), which has shown benefits in the staging and management of prostate and breast cancers. In the particular case of prostate cancer, WB-MRI and PET/CT with new tracers (Ga-prostate-specific membrane antigen [PSMA], 18F-fluciclovine, etc.) improve the assessment of both the extent of metastatic dissemination and the response to systemic treatments [21]. In breast cancer, there has also been an important improvement in staging with the advances in imaging techniques. A prospective comparison of CT and 18-F-FDG-PET/MRI in 80 patients with breast cancer showed that the latter was able to detect all 7 patients with distant metastases without any false-positive findings while CT missed metastases in 3 patients and provided false-positive findings in 3 patients [22]. However, no unique imaging strategy is consistently superior for the evaluation of metastatic bone disease in all tumor types and clinical scenarios.

The objectives of the treatment of patients with bone metastases include maximizing pain control, preserving and restoring function, minimizing the risk of SREs, stabilizing the skeleton if necessary and improving local tumor control. The current therapeutic options include pain management with analgesics, which can be administered in combination with (1) osteoclast inhibitors or bone modifying agents (including bisphosphonates and denosumab); (2) systemic cancer therapy (chemotherapy, immunotherapy, targeted therapies and hormone therapy); (3) radiotherapeutic treatment (external beam radiation therapy (EBRT), stereotactic body radiation therapy (SBRT)); (4) bone-targeting radiopharmaceuticals (e.g., samarium-153, strontium-89, and radium-223); (5) surgery, which is typically reserved for patients with a complete or imminent pathological fracture; and (6) local treatments, such as vertebroplasty, kyphoplasty, and image-guided thermal ablation [23, 24].

Treatment recommendations should be individualized according to the symptoms and impact on the quality of life of the patient, his or her functional status, the clinical status of the disease (extensive metastatic or oligometastatic disease), the estimated life expectancy, and whether there is an imminent or actual fracture in the affected bone [25].

To effectively manage patients with metastatic bone disease, it is essential to have consistent, reproducible, and validated methods to evaluate the response to treatment. The imaging criteria most commonly used are the Response Evaluation Criteria in Solid Tumors (RECIST) version 1.1. These and other similar classifications are predominantly focused on the physical measurement of solid tumors. A disease that cannot be easily measured with a ruler or calipers, as with most bone metastases, is designated nonmeasurable, and these patients are often not eligible for clinical trials, which may be the only available source of therapy. Nevertheless, current methods of evaluating therapeutic responses in cancer with bone metastasis remain problematic with wide variations in clinical practice. Among the currently available imaging modalities, WB-MRI and hybrid techniques combining morphological and functional data are the most sensitive and specific, and PET/CT and PET/MRI are becoming increasingly important in this regard [26, 27].

A group of experts selected and supported by the Spanish Society of Medical Oncology (SEOM), the Spanish Society of Medical Radiology (SERAM), and Spanish Society of Nuclear Medicine and Molecular Imaging (SEMNIM) met to discuss and provide an up-to-date review of our current understanding of the biological mechanisms through which tumors spread to bone and describe the imaging methods available to diagnose bone metastasis and monitor their response to oncological treatment, focusing on patients with breast and prostate cancer. To this end, the experts met in Madrid (Spain) to agree on the topics to be addressed in the consensus and distribute these topics among the experts. Afterward, all experts revised several drafts prepared until an agreement on the final version was reached.

## Biological mechanisms of bone metastasis or pathophysiology and molecular biology of bone metastasis

Several biological factors that predispose patients to bone dissemination in breast and prostate cancer have been described in recent years. When tumor cells escape the primary tumor, the majority do not survive in the bloodstream with only 0.02–0.1% of those that reach the blood being viable [1]. Tumor cells with positive expression of the chemokine receptor CXCR4 are mainly attracted to the bone stroma through the chemokine CXCL12. The high expression of adhesion molecules, such as integrins and VCAM-1, allows the binding of circulating tumor cells (CTCs) to the bone marrow (BM) stroma [7]. Once they reach this hematopoietic cell niche in the BM, tumor cells can undergo different dormancy periods. The BM microenvironment is characterized by low pH and oxygenation and elevated levels of extracellular calcium, which promote this dormancy and resistance phenomenon. At a certain time, dormant tumor cells transform into tumor-initiating cells (TICs) that begin the bone colonization process and produce an imbalance in the homeostatic mechanisms that regulate bone formation and resorption (bone turnover), ultimately leading to the establishment of metastasis [7, 28]. These regulatory mechanisms include the RANK/RANKL pathway, which coordinates the relationship between osteoblasts and osteoclasts. When these molecules bind to tumor necrosis proteins (tumor necrosis factor—TNF) through the expression of the osteoprotegerin (OPG) receptor, they stimulate bone resorption through osteoclasts, leading to lytic lesions. Overexpression of inhibitory factors of the Wnt pathway (such as DKK-1, among others) also promotes the formation of lytic lesions. In the case of blastic lesions, the Wnt/ET/BMP pathway is the main regulatory mechanism described. This pathway plays a crucial role in osteoblasts’ differentiation, function, and development [29, 30].

## Diagnostic tests for the initial diagnosis and staging of bone metastasis

Imaging modalities play a leading role in the early and accurate detection of bone involvement in the initial staging process, identifying complications, such as fractures or spinal cord compression, and evaluating response to local and systemic therapies.

The detection of metastatic bone disease rests on two possible foundations according to the imaging modality: the direct identification of the tumor and its tissue infiltration or the visualization of the bone reaction in the presence of tumor cells [31]. Notably, the biological basis of osteoblastic and osteolytic lesions is completely different. Bone resorption and bone formation are two intimately linked processes in health, but bone turnover is elevated and distorted in malignant lesions [32]. In this manner, osteoblastic metastases show a reduction in bone resorption and an increase in the stimulation of osteoblasts. On the other hand, osteolytic lesions show reduced osteoblastic activity and increased osteoclast stimulation.

Based on these foundations, anatomical and functional diagnostic tests have been developed that offer decisive and complementary information to classify patients as having advanced-stage cancer.

### Bone X-ray

The use of two orthogonal radiographic projections of an area that is painful or suspicious for an acute event in an oncological patient is, in many cases, the initial method for bone metastasis detection. Although it uses ionizing radiation, it is an inexpensive, fast and accessible exploration—that allows detection of the presence of lytic lesions (with greater than 50% destruction of the mineralized bone), blastic lesions, mixed lesions or complications, such as pathological fractures [33]. However, the use of the classical metastatic bone series to systematically exclude the presence of metastasis has not been recommended for years given its low diagnostic yield; thus, its role has been relegated to the study of doubtful or clinically significant areas.

### Computed tomography

Computed tomography (CT) is a primarily structural technique that provides a high spatial resolution and an adequate evaluation of the trabecular and cortical bone components. To detect lytic lesions, bone destruction of at least 20–30% is required. Therefore, the role of this technique in the detection of early metastatic bone disease is limited, especially in cases in which bone destruction has occurred in osteoporotic bone or bone with degenerative changes [34]. In addition, CT shows modest results in the detection of malignant infiltration of the bone marrow, making it difficult to differentiate between small metastases and normal fat bone marrow. For example, in a recent meta-analysis of the detection of bone metastases with various imaging techniques, the average sensitivity and specificity of CT were 73 and 95%, respectively. The sensitivity was significantly lower than those of bone scintigraphy, magnetic resonance imaging (MRI), and positron emission tomography (PET), although its specificity was greater than that of bone scintigraphy and similar to those of PET and MRI [35]. With the advent of dual and spectral CT, which has the unique ability to differentiate materials by their atomic number, new perspectives have been opened in assessing metastatic bone disease as they allow a better assessment of bone composition. Preliminary results have shown similar capabilities of dual energy CT to bone scintigraphy in the detection of bone metastases [36]. Finally, a recent report has shown that spectral CT and WB-MRI equally perform in detecting breast cancer metastases on a per-patient basis, and are superior to conventional CT. Specifically in detecting of metastatic bone lesions on a per-lesion basis, there were non-significant differences in sensitivity and specificity between spectral CT and WB-MRI, although, WB-MRI showed a significant superior area under the curve (AUC) [37].

### Diffusion-weighted whole-body magnetic resonance imaging

MRI is a technique that classically provides structural information with excellent average sensitivity and specificity in detecting of bone metastases equivalent at least to those of 18-FDG-PET/CT due to its excellent tissue contrast [35]. MRI allows the adequate detection and characterization of any bone metastasis, including lytic, blastic or mixed metastases, and has the advantage of not using ionizing radiation. MRI is also the technique of choice to detect complications of metastasis, such as pathological fractures or spinal cord compression in the case of vertebral lesions, allowing an accurate evaluation of the extension to adjacent structures and the possible infiltration of soft tissues or neurovascular bundles. In addition, it is an excellent technique for the early detection of bone marrow infiltration, which precedes the morphological changes produced by bone metastases and, therefore, for the early detection of metastatic bone disease [38].

Using state-of-the-art equipment, recent technological advances have allowed performing whole-body studies that cover the axial skeleton or even the extremities in examination times of less than 30 min. Traditional morphological sequences can be complemented with functional information provided by techniques, such as perfusion, proton spectroscopy or diffusion, that quantitatively assess tumor angiogenesis and permeability, metabolism and cellularity, respectively [39]. Diffusion, which assesses the Brownian motion of free water, allows the quantitative determination of the degree of tissue interstitial occupation and indirect estimation of the cellularity of a tissue based on the apparent diffusion coefficient (ADC). The diffusion-weighting is controlled by the b value, a factor that reflects the strength and timing of the gradients during the imaging acquisition. In addition, DWI is the only functional or molecular MRI technique that can be applied to whole-body (WB)-MRI studies.

State-of-the-art WB-MRI protocols (Table 1) should include T1- and T2-weighted morphological sequences without and with fat suppression in addition to diffusion-weighted sequences and should always be interpreted jointly. In addition, if T1- or T2-weighted Dixon sequences are used, they allow differentiation and quantification of the fat and water components of the bone marrow, allowing a better understanding of the changes produced in the bone marrow with different therapies [32]. The multiparametric information derived from WB-MRI has shown global results similar to those for 18-Fluorodeoxyglucose (^18^FDG)-PET/CT in detecting of metastasis, although WB-MRI is considered superior in evaluating of hepatic and cerebral secondary involvement. However, it has shown more limited results than PET in the evaluation of pulmonary and lymph node metastases. In the specific case of bone metastases, diffusion-weighted WB-MRI is considered the whole-body imaging technique with greatest diagnostic accuracy for their evaluation, with a greater performance than scintigraphy or PET/CT [40].

**Table 1:** Recommended protocol for diffusion-weighted WB-MRI for the evaluation of bone metastases

Region	Sequence	Plane
Head to thighs	STIR	Coronal
Head to thighs	Dixon TSE T1	Coronal
Head to thighs	TSE T2	Axial
Head to thighs	Dixon GE T1	Axial
Head to thighs	Diffusion-weighted imaging	Axial or coronal
Spinal column	STIR	Sagittal
Spinal column	TSE T1	Sagittal

*STIR* short-tau inversion-recovery, *GE* gradient echo, *TSE* turbo spin echo

A meta-analysis published in 2020 confirmed that WB-MRI was superior to bone scintigraphy in the detection of bone metastasis with higher sensitivity (94 vs. 80%, respectively) and diagnostic accuracy and higher but comparable patient-based specificity (99 vs. 95%, respectively) [41]. Similarly, the SKELETA prospective clinical trial evaluated 26 patients with breast cancer and 27 with prostate cancer (PCa) with a high risk of metastatic bone disease using bone scintigraphy, single photon emission computed tomography (SPECT), SPECT/CT, 18F-sodium-fluoride (^18^F-NaF)-PET/CT and diffusion-weighted WB-MRI. The areas under the curve (AUCs) for the detection of bone metastases by SPECT/CT, ^18^F-NaF-PET/CT and diffusion-weighted WB-MRI were significantly higher than those of bone scintigraphy and SPECT. Additionally, the sensitivity and AUC in the lesion-based analysis were significantly higher for ^18^F-Na F-PET/CT and diffusion-weighted WB-MRI compared with the remaining techniques. In addition, similar results were noted between the two techniques despite fewer equivocal lesions for WB-MRI [42]. In addition, another recent meta-analysis evaluating the detection of bone metastases in PCa showed superior results for WB-MRI (sensitivity 0.95, specificity 0.96, and AUC 0.98) compared to bone scintigraphy (sensitivity 0.79, specificity 0.82, and AUC 0.88) and choline-PET/CT (sensitivity 0.87, specificity 0.97, and AUC 0.95) [43]. Furthermore, a recent study compared the performance of diffusion-weighted WB-MRI to FDG-PET/CT in 39 patients with 239 bone metastases, and the sensitivity, specificity, overall accuracy, positive predictive value, and negative predictive value were 93.0%, 87.8%, 89.6%, 79.8%, and 96.0%, respectively, for WB-MRI and 92.5%, 92.0%, 92.1%, 85.7%, and 95.9%, respectively, for FDG-PET/CT. The specificity of WB DWIBS in detecting bone metastases was significantly lower than that of FDG-PET/CT (*p* < 0.05), whereas the remaining results were not significantly different between the two techniques [44]. Finally, another meta-analysis that evaluated the detection of bone metastases from different malignant tumors showed a sensitivity and specificity greater than 90% for diffusion-weighted WB-MRI; however, the specificity for diffusion-weighted WB-MRI was lower than that for WB-MRI including only morphological series [45]. Therefore, diffusion-weighted WB-MRI has been proposed as the technique of choice to evaluate patients with bone metastases of an unknown primary tumor location [46]. In the case of bone metastases, the objectives of whole-body imaging tests for staging go beyond determining the existence of metastatic disease as it is also critical to detect disease sites and quantify disease volume. In some tumors, such as PCa, the metastatic tumor load has a high prognostic value and is critical for determining therapeutic options as well as confirming or excluding the presence of oligometastatic disease (≤ 5 metastases) as it allows more aggressive selective treatments, such as targeted radiotherapy, surgery or ablative procedures [27].

Today, WB-MRI is considered a first-line technique in the search for distant metastases of breast, prostate, and neuroendocrine tumors and in the extension study of multiple myeloma and in the evaluation of pediatric and pregnant patients with cancer [40]. WB-MRI is also considered a valid alternative in the distal staging of melanoma, lung cancer, thyroid cancer, renal cancer or colon cancer. Especially interesting is the use of MRI to perform single-step local and distal staging of gynecological, rectal and prostate tumors. Specifically regarding PCa, according to current clinical guidelines, it is only recommended to use imaging tests to exclude metastatic disease in patients with untreated PCa with an unfavorable intermediate-to-high risk of locally advanced disease and in cases of suspected relapse after treatment [12, 47]. In this clinical scenario, WB-MRI has been proposed by several authors as an alternative to the current imaging algorithm for regional and distal staging, which includes the use of contrast-enhanced CT and bone scintigraphy. WB-MRI has demonstrated equivalent sensitivity and specificity in the detection of lymph node metastases to CT and superior results compared to bone scintigraphy for the detection of bone metastasis, allowing the avoidance of the use of two different tests with ionizing radiation and two different injections of intravenous contrast [48].

### Bone scintigraphy

Bone scintigraphy (BS) with technetium-labeled diphosphonates (^99m^Tc-HDP) continues to be the technique of choice and the most widely used in the evaluation of osteoblastic or mixed metastatic bone disease [27, 49]. The accessibility and low cost of BS and its capacity to detect osteogenic activity up to 2–18 months before the appearance of clinical, analytical or bone X-ray signs, have made this technique an essential tool in the study of initial staging.

^99m^Tc-HDP is a calcimimetic radiopharmaceutical; its uptake in bone is influenced by local blood flow, bone remodeling activity, and extraction efficiency. Once deposited in the bone, diphosphonates are absorbed by hydroxyapatite crystals on bone mineralization surfaces [50]. A change of only 5–10% in the lesion/healthy bone ratio is sufficient to induce pathological deposition of the radiopharmaceutical [23].

As a whole-body study, bone scanning allows the visualization of the entire skeleton in a single image, making it possible to detect appendicular lesions that are usually not included in conventional studies. Another advantage lies in its reasonably high global sensitivity (78%, which can reach 87% if SPECT tomographic acquisition is used) [26]. However, such SE leads to the detection of benign lesions with intense osteogenic activity, such as eosinophilic granulomas and fibrous dysplasia or enchondromas, as causes of false positives and low specificity in the oncological context [51]. In addition, posttraumatic bone changes and a mechanical or degenerative etiology also hinder the interpretation of scintigraph images.

In addition to these weaknesses, this technique also has limited spatial resolution, especially when only a planar image is used [52], and a practical inability to detect purely lytic lesions, which usually have a more aggressive tumor biology, in which repairing perilesional bone remodeling is not present, being the new bone formation tissue the substrate for the deposition of the labeled diphosphonates. Finally, diffuse metastatic bone dissemination with a “superscan pattern” can go unnoticed by inexperienced eyes, which would lead to incorrect disease staging and management.

SPECT imaging and, more recently, hybrid SPECT/CT imaging have been included in routine clinical practice to minimize the weaknesses of the planar image (global sensitivity and specificity of SPECT for the detection of bone metastases of 87 and 91%, respectively), allowing the evaluation of the bone structure in the three planes of space through simultaneous visualization of the anatomical image and functional or bone metabolism image [26, 53].

### Positron emission tomography

There are three classes of PET radiopharmaceuticals used to obtain bone images: ^18^F-sodium fluoride, which is an osteotropic or calcimimetic element, is a specific bone marker with affinity for osteoblast activity and fixation mechanism similar to that of ^99m^Tc-HDP [54]; specific oncotropic markers, such as ^68^ Ga-HER-2 or ^18^F-fluoroestradiol in the case of breast cancer or fluoromethylcholine or prostate-specific membrane antigen (PSMA) as membrane-specific antigens in the case of prostate cancer; and metabolic tracers or nonspecific oncotropic markers, such as [^18^F] 2-fluoro-2-d-glucose (FDG) as a marker of tumor glycolytic activity [55].

The utility and applicability of PET imaging lie in the radiopharmaceutical used, among other factors; however, in general, PET has a much higher spatial resolution than conventional scintigraphy images [53].

PET/CT with choline analogs has shown higher sensitivity and specificity in detecting prostate bone metastases than BS, which can be explained by the optimal detection of bone marrow involvement and its ability to monitor cell proliferation compared with ^18^F-NaF-PET [56].

Lipid metabolism in bone metastases varies according to the type of lesion; thus, less sclerotic lesions exhibit greater metabolic activity. These findings led to the establishment of the hypothesis that hypometabolic sclerosing lesions reflect reparative bone changes, whereas hypermetabolic lesions with no significant increase in radiological density are related to spinal or microsclerotic metastases in which cellularity and tumor replication, generally present in the early stages of the lesion, are greater than reparative bone activity [57].

More recently, PET/CT with PSMA, labeled with ^68^ Ga or ^18^F, has shown a higher SE in the detection of bone disease [58–60]; however, there is little evidence of its yield. The most relevant study in which PSMA-PET/CT or PET/MRI was compared with BS in 37 patients showed sensitivity values of 100 and 57% for ^68^ Ga-PSMA-PET/CT and BS, respectively, with comparable specificity values (100 vs. 96%, respectively) [61]. Table 2 shows the most important differences between the main PET radiopharmaceuticals used in clinical practice.

**Table 2:** Positive and negative aspects of the main PET radiopharmaceuticals used in clinical practice for the detection of metastatic bone disease

Radiopharmaceutical	Target	Strengths	Weaknesses
^18^F-FDG	Cellular glucose metabolism	Glycolytic activity allows identification of lesions with aggressive tumor biology	Low diagnostic yield in prostate cancer due to low affinity for glucose
^18^F-Choline	Cellular membrane proliferation	Allows the identification of bone and soft tissue lesions	Limited sensitivity for the detection of liver lesions
^68^ Ga or ^18^F-PSMA	Cell membrane protein expressed in prostate carcinoma	Identifies bone lesions and lymph node involvement at less elevated PSA values and PSA doubling time	Little bibliographic evidence on the real impact on survival, PSMA expression is increased in patients on antiandrogenic therapy
^18^F-NaF	Bone remodeling	High sensitivity for the detection of bone metastases	Does not allow visualization of extraosseous lesions

^*18*^*F-FDG* [18F] 2-fluoro-2-d-glucose, ^*18*^*F-NaF* 18F-sodium-fluoride, *PSA* prostatic specific antigen, *PSMA* prostate-specific membrane antigen

#### ^***18***^***F-FDG-PET***

^18^F-FDG is the most universally widespread oncotropic radiopharmaceutical; its deposition is related to tumor glycolytic activity, making it a direct measure of cell viability and tumor cell biology. Its high availability and its ability to quantify metabolic activity as an indispensable parameter for the evaluation of the response to therapy are its main advantages [26, 62]. However, this imaging modality is not without disadvantages, which are especially evident in certain scenarios. The tumor affinity for FDG and, therefore, the diagnostic yield and SE of the test depend on the tumor histological type in many cases [63]; thus, well-differentiated neoplasms and indolent tumors with a low mitotic rate or low aggressiveness can go unnoticed with FDG-PET imaging. Similarly, sclerosing bone metastases tend to have a lower glycolytic rate than purely lytic lesions or mixed lesions presumably due to the greater aggressiveness of the latter. In any case, a global sensitivity and specificity for detecting bone metastases of 98 and 56%, respectively, have been reported [26]: the specificity increases to 97% when combined with CT in hybrid PET/CT images [64].

Based on current evidence, routine FDG-PET/CT imaging is not recommended in patients with early-stage breast carcinoma (I or II) unless there are signs or symptoms of distant disease [65–68]; however, there is a sufficient level of evidence that supports its use in the initial staging of advanced-stage disease (level of evidence IA) based on an improvement in regional and distal staging.

#### ^***18***^***F-NaF-PET***

The main osteotropic radioisotope used for the acquisition of PET images is ^18^F-NaF, which has a higher extraction rate than ^99m^Tc-HDP, with threefold higher plasma extraction in bone metastases compared with adjacent normal bone. After diffusion through the capillary wall into the extracellular space, the fluoride ions undergo a gradual exchange with the hydroxyl groups of hydroxyapatite crystals to form fluorapatite and then deposit on the bone surface where remodeling is maximal [26].

Compared to other functional imaging modalities, NaF-PET has demonstrated a sensitivity and specificity clearly superior to those for bone scanning with ^99m^Tc-HDP for the detection of metastasis, especially of the lytic type [35, 63], and superior to that for FDG-PET, especially for sclerotic lesions [69]. Global sensitivity and specificity values of 100 and 97%, respectively, have been reported if hybrid PET/CT images are used [26].

The weaknesses of NaF-PET are similar to those of bone scanning and are mainly based on the high increase in bone remodeling in certain benign lesions and posttraumatic and infectious lesions.

## Role of imaging techniques in the therapeutic monitoring of bone metastasis

- 1. Evaluation of the response of bone metastases in breast cancer

Bone metastases are the most common metastases in breast cancer. These metastases are most often osteolytic, but blastic metastases predominate in up to 20% of cases. Each predominant pattern is related to a different molecular mechanism at the level of the tumor cells in the bone marrow. Although it may be the initial form of presentation, the mixed pattern is typically more frequently observed during therapeutic follow-up when sclerosis of the metastases occurs with good response to treatment. There are no clinical, analytical or imaging diagnostic methods that predict which patients will develop metastatic bone disease; therefore, in late detection, the disease volumes and mutational heterogeneity are greater, requiring more toxic systemic therapies than in earlier disease stages [49]. Additionally, a greater metastatic bone disease volume is associated with reduced overall survival [70]. In addition, current clinical guidelines specify few recommendations for detecting of bone metastases in patients with advanced breast cancer and do not recognize the poor sensitivity of bone scintigraphy and CT [71, 72].

### Computed tomography

In general, the role of CT in the therapeutic monitoring of bone metastases is controversial. Bone metastases confined to bone are not considered measurable target lesions for RECIST unless there is a measurable soft tissue component [73]. Therefore, RECIST v1.1 shows limitations in the determination of the presence of bone metastasis and in the quantification of tumor load. In the specific setting of follow-up of breast cancer metastases, although CT is used in many clinical trials to assess response to treatment where there is a measurable soft tissue component, this technique shows important limitations [49]. In general, in most of the bone metastases imaging response criteria, and specifically in the one developed at the MD Anderson Cancer Center, the development of sclerosis in bone metastases is considered to be a sign of response to treatment [74]. Therefore, these same criteria do not consider that there is a progression in the case of the development of new osteosclerotic lesions if there are no other associated signs of progression. Specifically, in breast cancer bone metastases, lytic changes suggest that the progression and development of sclerosis is linked to improvement of metastatic disease, but sclerosis can also be found in cases of progression [75]. In addition, the MD Anderson Cancer Center criteria do not include references for tomodensitometry measurements of bone lesions; therefore, patients under treatment with bisphosphonates cannot be assessed by these criteria, as this kind of therapy suppresses osteoclast activity and increases bone mineral density. Finally, CT does not assess the heterogeneity of the response and shows limitations in the evaluation of early responses or resistance to treatment.

The use of dual or spectral energy CT allows the generation of a bone-iodine separated set of images and can be used to detect bone metastases with greater sensitivity [36] and better characterize bone lesions in oncological patients [76]. However, data on its potential role in therapeutic follow-up are lacking. Furthermore, the use of bone subtraction maps has been proposed to increase the accuracy and efficiency of CT in the therapeutic monitoring of pelvic breast cancer metastases [77].

### Diffusion-weighted WB-MRI

The follow-up of patients with bone metastases undergoing treatment is typically performed with CT, ^18^FDG PET/CT or bone scintigraphy. CT and bone scintigraphy only allow assessing whether patients are stable or have worsened but not whether they have improved in an objective and quantifiable way [32]. Criteria for progression and regression of bone metastases were defined with morphological MRI sequences [78] (Table 3). However, there are few clinical data supporting the use of structural sequences to evaluate therapeutic responses. Other limitations in the assessment of responses with morphological sequences are related to the presence of sclerosis, fibrosis or diffuse bone necrosis that usually occurs after several treatment cycles, making it difficult to determine the presence of new lesions and changes in the lesions that are being treated. Another confounding factor is the phenomenon of pseudoprogression in T1-weighted sequences, which produces a diffuse decrease in the bone signal due to diffuse bone edema secondary to massive cell death and inflammation, which can be confused with progression [49].

**Table 3:** MRI morphological criteria of bone metastasis progression and regression

Progression criteria	Regression criteria
New focal lesion or area of diffuse metastatic infiltration in the bone marrow	Disappearance of a focal lesion or area of diffuse metastatic infiltration in the bone marrow
Increase in the size or number of focal lesions	Decrease in the size or number of focal lesions
Evolution of focal lesions to a diffuse neoplastic pattern	Decrease in the extension of a diffuse neoplastic pattern
	Appearance of intra- or peritumoral fat (fat dot and halo signs)
	Decrease in contrast enhancement
	Development of dense sclerosis with well-defined or thin margins
	Disappearance of the hyperintense perilesional ring in fat-suppressed T2-weighted sequences

**Fig. 2:**
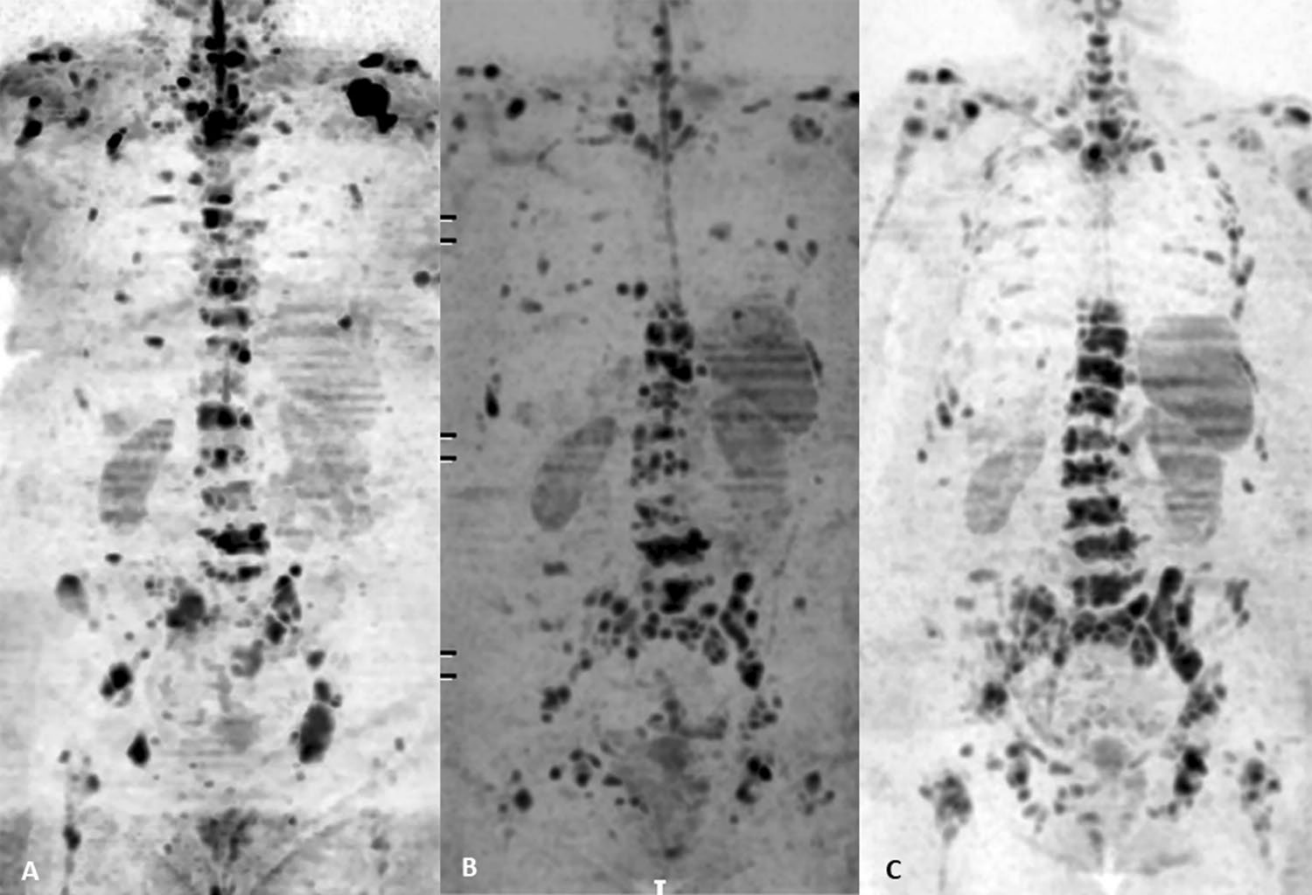
A 77-year-old patient with breast cancer presented with diffuse bone metastatic involvement in the initial staging with diffusion whole-body MRI (**A**, left) with a heterogeneous response at 6 months (**B**, center) given progression of pelvic, lumbar, and lower dorsal and costal bone disease and partial response of metastatic disease in middle and upper dorsal bodies and shoulder girdle. At the 3-month follow-up (**C**, right), we found a marked progression of bone disease of the generalized form

In the evaluation of therapeutic responses, the use of diffusion-weighted sequences and ADC quantifications is much more interesting. An appropriate therapeutic response is generally associated with an increase in the ADC related to cytotoxicity, decreased cellularity and loss of cell membrane integrity. However, this response may vary depending on the type of therapy and the time since the start of treatment [32] (Table 4). ADC measures have shown adequate repeatability and allow the detection of variations greater than 12% related to treatment; therefore, they are an adequate biomarker to detect the changes produced by therapy [79]. In addition, WB-MRI, particularly the diffusion-weighted sequence, allows determination of the tumor load and the individual changes induced by therapy through ADC measures. This information allows differentiation between stable, responsive and nonresponsive patients, including in the final stages of the disease when the aforementioned fibrotic changes occur in the bone marrow (Fig. 2). Additionally, DWI allows the evaluation of heterogeneous response patterns to treatment, which are usually produced when the metastases are derived from different cell clones [32].

**Table 4:** Patterns of responses to therapy for bone metastases evaluated with diffusion-weighted sequences and apparent diffusion coefficients

Response patterns	Meaning
Increase in the volume, appearance of new areas or increase in the signal intensity of abnormal areas with high *b* values	Progression
The ADC may increase, remain stable or show a slight decrease	
No changes in lesions with high *b* values	Response
Marked ADC increase (T2 shine-through)	
Decrease in the signal intensity in abnormal areas with high *b* values	Response
Significant increase in the ADC: varies according to the type of treatment and changes that occur in the bone marrow	
No changes in lesions with high *b* values	Stability
ADC stable or slight (not significant) decrease or increase	
Decrease in the signal intensity of lesions detected with high *b* values	Undetermined^a^
Decrease in ADC	

*ADC* apparent diffusion coefficient

^a^This pattern is usually visualized when sclerosis or myelofibrosis develops in the bone marrow or when there is an increase in the fatty bone marrow. This pattern is more common in responders but can occur in cases of sclerotic progression. When it occurs, the response must be evaluated based on morphological sequences

Among WB imaging techniques, diffusion-weighted WB-MRI offers the greatest capabilities for evaluating the therapeutic response of bone metastases from breast cancer. This is the only technique along with ^18^FDG-PET/CT that allows direct evaluation of the viability of tumor cells in the bone marrow. Clinical guidelines recommend the use of clinical assessment, tumor markers and CT and bone scintigraphy as imaging methods and only recognize an optional role for ^18^FDG-PET/CT; they do not consider WB-MRI [71, 80]. However, WB-MRI with morphological sequences, such as ^18^FDG-PET/CT, presents problems in detecting tumor activity in bone if a healing process has developed in the bone marrow via previous therapies, if there is bone marrow hyperplasia (for example, by colony-stimulating factors) or if there is sclerotic progression. In this sense, recent data support the reliability of morphological MRI sequences to determine whether there is progression or stability; however, there are limitations to determining whether there is a disease response [81]. However, these limitations can be overcome with the use of diffusion-weighted sequences with ADC measures, as demonstrated by another recent series that compared the efficacy of CT and diffusion-weighted WB-MRI for the evaluation of systemic response to therapy in 101 patients with breast cancer with bone metastases. WB-MRI detected additional sites of distant disease in 53.3% of the patients. In addition, WB-MRI classified 65.5% of cases as a progression that were labeled stable disease on CT, mainly in patients under treatment with second-line drugs. Therapy was modified in 35% of the cases due to diffusion-weighted WB-MRI findings [82]. Finally, a recent prospective study in 45 patients with bone-only metastatic breast cancer confirmed the superior performance of WB-MRI in therapy monitoring, as progressive disease was evident in only half of participants at bone scintigraphy compared to WB-MRI, and it also enabled identification of progressive disease before CT in most patients [83].

Therefore, diffusion-weighted WB-MRI provides the opportunity for early detection of the presence of distant bone disease, allows assessing the heterogeneity in response to targeted therapies and identifies patients who do not benefit from a line of treatment at an early stage after its establishment [32]. Although sufficient data to systematically endorse the use of diffusion-weighted WB-MRI in the management of breast cancer is lacking, its use in certain scenarios is beneficial (Table 5).

**Table 5:** Potential clinical scenarios in breast cancer patients where diffusion-weighted MRI could be applied

Equivocal findings in scintigraphy or CT
Differentiation between metastatic and osteoporotic fracture
Exclude bone metastases in patients with low suspicion of bone disease
Staging of patients with high-risk breast cancer at diagnosis^a^
Substitute for CT or ^18^FDG-PET/CT in patients with early locoregional recurrence
Determining the extent of the disease in therapy monitoring
Detection of oligometastatic disease that is a candidate for local treatment
Evaluation of locally advanced breast carcinoma
Evaluation of local recurrence
Assessment of metastatic bone disease and/or hepatic disease treated with systemic therapy

*CT* computed tomography, ^*18*^*FDG-PET/CT* 18-fluorodeoxyglucose-positron emission tomography/computed tomography

^a^High-risk patients are considered those with inoperable locally advanced breast cancer, inflammatory cancer, breast cancer diagnosed during pregnancy, cancer with more than four positive nodes, or triple-negative breast cancer

### Bone scintigraphy

BS with ^99m^Tc-HDP is indicated and included in the different clinical guidelines in the initial staging of breast carcinoma from stage IIb (level of evidence 2B) and whenever there is clinical justification regardless of the stage [84–86]. However, after the onset of systemic therapy, changes in response may not be detectable by scintigraphy or may take up to 6 months to be reflected in approximately 50% of responding patients [49, 87]. This finding is attributed to the fact that scintigraphy manifests peritumoral bone structural alterations but does not directly represent tumor cellular activity. A paradoxical response defined as a “flare” phenomenon, which is characterized by a greater osteoblast reaction in known lesions or even the appearance of “new lesions”, can also be observed after therapy as a translation of reparative osteogenic response in preexisting lesions that are predominantly lytic. Finally, in some cases, a decrease in uptake can be observed in rapidly progressive disease due to massive destruction without reparative osteoblastic activity, but this response is uncommon. This decrease in uptake intensity can be misinterpreted as a response to treatment [88].

In addition, certain chemotherapy regimens can directly interfere with bone remodeling. For example, denosumab, an anti-RANKL monoclonal IgG2 antibody, inhibits osteoclast function [89], interfering with the evaluation of response in bone scans and simulating a correct response of metastatic disease.

Certain limitations of scintigraphic images could be partially solved when evaluating the response to treatment by applying the most recent technological advances, such as the quantification of tumor load (percentage of total skeletal mass), quantitative normalization of the uptake intensity and segmentation of bone uptake compared to healthy controls [89]. However, this technology is not yet applicable in daily clinical practice. Similarly, hybrid SPECT/CT imaging facilitates the characterization of doubtful lesions in planar imaging as well as the identification of lytic lesions and their sclerotic transformation in responding patients.

In any case, the United Kingdom’s National Institute for Health and Clinical Excellence (NICE) has discouraged the use of BS as a tool for the evaluation of responses: "there is no evidence that bone scintigraphy can be used to evaluate the response to treatment" [84]. Therefore, its use in this scenario is not recommended.

BS should be used only when there is suspicion of progression, in which case 2 new lesions should be documented after the “flare-up” period (first 12 weeks) with a lapse of at least 6 weeks [26, 49, 89]. BS should not be used for the assessment of response to treatment due to the risk of erroneous image interpretation.

The specific bone response criteria proposed by MD Anderson Cancer Center in 2004 combine radiological findings (conventional radiography, CT, and MRI) with BS findings. The criteria propose response stratification through qualitative evaluation of scintigraphy. Thus, complete normalization of the uptake of labeled diphosphonates translates into a complete response, a subjective decrease in uptake ≥ 50% into a partial response, and a subjective increase ≥ 25% or the appearance of new lesions into disease progression [90].

### Positron emission tomography

#### ^***18***^***F-NaF-PET***

Although the absorption of ^18^F-fluoride depends on regional blood flow and osteoblastic activity, similar to ^99m^Tc-HDP, the increased spatial resolution of PET imaging, higher lesion/background uptake ratio and better visualization of lesions located in bone marrow make ^18^F-NaF-PET a more sensitive bone imaging modality for the detection of metastatic, blastic and even lytic lesions and for the evaluation of response to treatment [31, 91]. However, its reduced availability and increased cost hinder its use in routine clinical practice.

#### ^***18***^***F-FDG-PET/TC***

As an oncotropic radiopharmaceutical, ^18^F-FDG provides direct information on tumor cell viability in metastatic lesion beds. However, the SE of the technique for the detection of secondary bone disease is directly influenced by certain factors specific to tumor biology, especially the immunohistochemical phenotype. Thus, semiquantitative metabolic parameters, such as the standardized uptake value (SUVmax), are clearly superior in biologically more aggressive neoplasias (high-grade, negative for hormonal receptors, HER2-positive or triple-negative cancers) compared with those that exhibit less aggressive tumor biology [92, 93].

Some chemotherapeutic agents produce cytostatic rather than cytolytic effects in tumor cells, controlling the disease without an apparent reduction in the size of the lesion and hindering the assessment of response based exclusively on morphological or volumetric criteria. The regular and well-defined tumor margins necessary for a reproducible radiological measurement are of less importance in functional imaging [94].

**Fig. 3:**
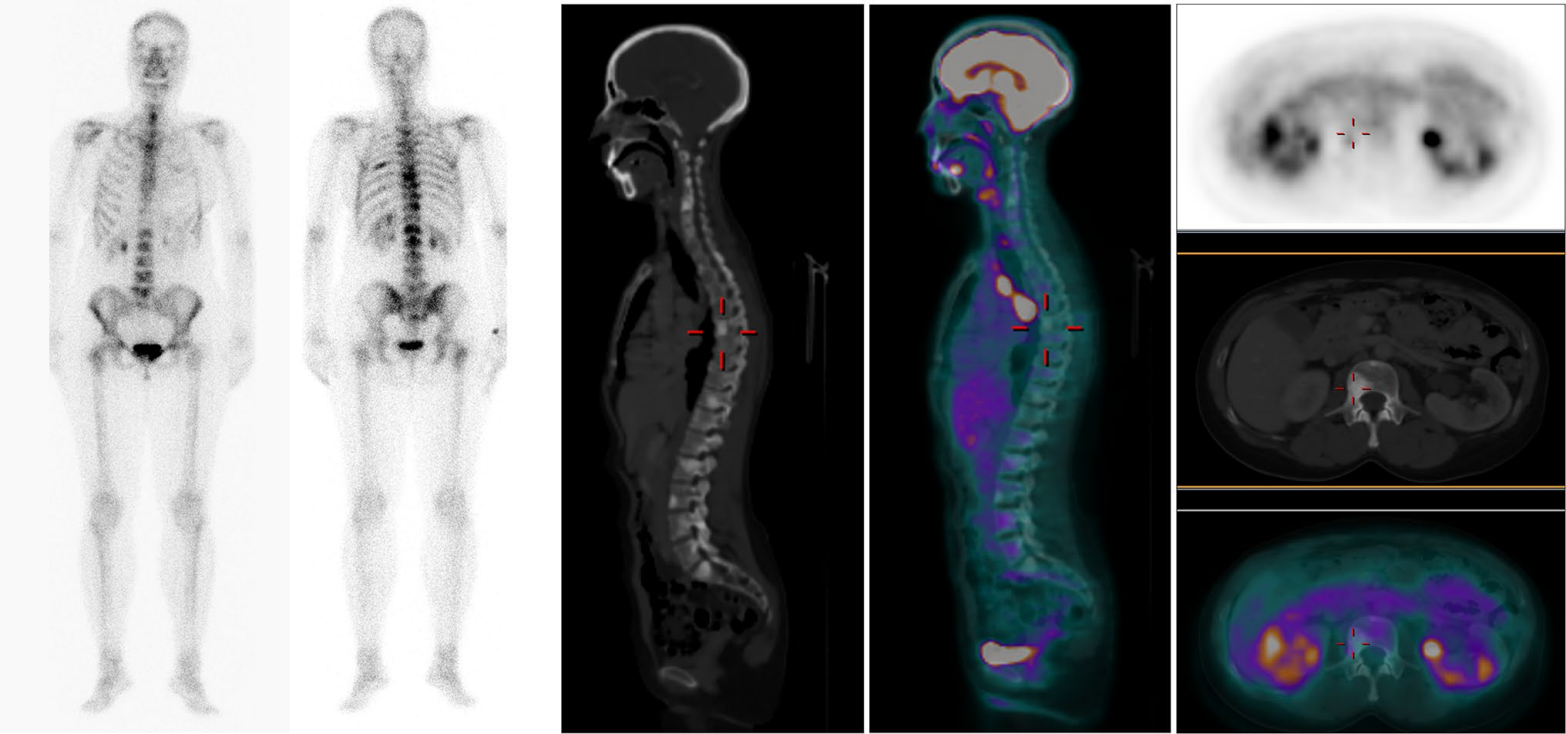
Added value of PET/CT in the characterization of treated secondary bone lesions. A 53-year-old woman, diagnosed with infiltrating ductal breast carcinoma, treated with surgery, chemotherapy, and radiotherapy. Bone scintigraphy and CT showed osteoblastic lesions suspicious for metastases that did not respond to treatment. In this scenario, FDG-PET/CT image makes it possible to rule out signs of tumor viability in skeletal lesions, classifying them as old lesions with a complete response to treatment

After initiating systemic therapy, FDG-PET can only be useful in evaluating response in patients with previous positive metabolic studies. Although it is not systematically indicated in the current international guidelines to evaluate the response of metastatic disease, an important potential advantage has been demonstrated over conventional imaging, with an excellent symptom/image concordance rate in patients with disease resolution or improvement [95] (Fig. 3). Although the persistence of focal hypermetabolism can demonstrate residual uptake after effective therapy that is probably related to macrophage activity, this feature often translates to uncontrolled disease. In contrast, the resolution of glycolytic activity indicates disease control, regardless of the appearance of the lesion on BS (often persistently positive) or its radiological appearance on the CT component of PET/CT [88].

Another scenario in which hybrid images can clarify the situation of metastatic disease is the "flare" reaction, which is observed as a generalized increase in the metabolic activity in the bone marrow after the use of granulocyte colony-stimulating factor and is very intense in some cases. This confounding factor in PET images has been described 7–10 days after initiation of tamoxifen or fulvestrant therapy for breast cancers with positive hormone receptors, which may indicate the success of the therapy [96].

Different quantitative criteria have been designed to assess the response to systemic therapy, such as those of the European Organization for Research and Treatment of Cancer (EORTC) and the more recent and standardized PET Response Criteria in Solid Tumors (PERCIST), which allow stratification of the response according to the percentage change in activity in the most active lesion between the pre- and post-treatment studies (Table 6).

**Table 6:** Criteria for the assessment of metabolic response

	EORTC	PERCIST
Target lesion	The defined pretreatment tumor region must correspond to the increased uptake of ^18^F-FDG (viable tumor)	Those who present an SULpeak of at least 1.5 times that calculated in the liver. If there is liver pathology, then it must be at least two times higher than a vascular ROI in the thoracic descending aorta Quantification of five measurable target lesions (no more than two lesions per organ)
Complete metabolic response	Normalization of the uptake of ^18^F-FDG in the tumor volume with indistinguishable activity of the surrounding healthy tissue	Complete disappearance of the pathological uptake of ^18^F-FDG in all lesions (target and nontarget) that have activity lower than hepatic activity and are indistinguishable from the vascular background Verification in 1 month if there is anatomical criterion of progression
Partial metabolic response	Reduction of between 15 and 25% of the SUV of the tumor after 1 chemotherapy cycle and > 25% after more than 1 cycle	Reduction of at least 30% in SULpeak in target lesions Verification in a follow-up study if there is an anatomical criterion of progression
Stable disease	Increase < 25% or reduction < 15% in SUV without an increase in the extent of uptake (< 20% of the largest dimension)	No complete or partial metabolic response or metabolic progression
Metabolic progression	SUV increase > 25%. Increase > 20% in the extension of uptake. Appearance of new pathological uptake of 18F-FDG	Increase in SULpeak > 30% in target lesions. Increase in the extent of uptake (> 75% in total lesion glycolysis, or TLG). Emergence of new pathological uptake of 18F-FDG not explainable by treatment or infection effects Verification in a follow-up study if there is an anatomical criterion of partial or complete response

*EORTC* European Organization for Research and Treatment of Cancer, *PERCIST* Positron Emission tomography Response Criteria In Solid Tumors, *ROI* region of interest, *SUV* standardized uptake value, *SUL* standardized uptake value corrected for lean body mass, *18F-FDG* [18F] 2-fluoro-2-d-glucose

Although an almost perfect correlation between both response criteria has been demonstrated [97], the EORTC uses SUVmax as a variable, whereas PERCIST proposes the use of the SUV corrected for lean body mass (SULpeak) as a normalization tool to decrease fat tissue as a confounder [98, 99].

The modified RECIST v1.1 criteria consider the metabolic behavior of the lesion as one of the variables of interest; however, this is only considered for the detection of tumor progression providing that the findings in functional images accompany a radiological correlate or show metabolic progression over time or are demonstrated in a subsequent radiological study [73].

### Specific radiopharmaceuticals for breast cancer

Specific oncotropic radiopharmaceuticals, such as ^18^F-fluoroestradiol (FES-PET), have demonstrated the ability to identify patients responding to treatment earlier than FDG-PET only 7–10 days after starting treatment with tamoxifen; these processes also facilitate correct selection of candidate patients for treatment. The use of FES-PET can increase the overall response rate from 23 to 34%. For patients with HER2/neu-negative breast carcinoma, the response to treatment can increase from 29 to 46% if the patient selection has been performed with FES-PET [55].

Specific radiopharmaceuticals for human epidermal growth factor 2 (HER2) allow the characterization of lesions by determining the expression of HER2 in the primary lesion and metastatic lesions with PET and facilitating the correct selection of candidate patients for targeted therapy. Similarly, radiopharmaceuticals for radiometabolic therapy directed against HER2 (theragnosis) have been designed and applied in the preclinical setting [100, 101].

- 2. Evaluation of the response of bone metastases in prostate cancer

Bone metastasis occurs in up to 62% of patients with newly diagnosed metastatic PCa and in greater than 40% of patients with castration-resistant prostate cancer (CRPCa) [27]. In addition, the volume of metastatic bone disease is an independent prognostic factor in patients with PCa [102]. Furthermore, it is especially important to detect oligometastatic disease (in initial staging, recurrence or progression), which is defined as five or fewer metastatic lesions (intermediate situation between localized and disseminated disease), because this scenario may require radical locoregional management, allowing the prevention of or delay in the spread of the disease and initiation of a specific therapy.

The current diagnostic pathway for metastatic PCa is based on the presence of symptoms, prostate-specific antigen (PSA) levels and the combination of scintigraphy and CT. Thus, in the castration-sensitive phase, the assessment is based on the presence or absence of symptoms and PSA levels. The role of imaging is typically focused on determining the initial tumor load and its changes in cases of response or progression. However, in CRPCa, imaging follow-up with the use of new therapies is recommended. For example, between 21 and 30% of patients under treatment with abiraterone or enzalutamide without clinical signs or elevated PSA levels develop radiological progression, and 40% of patients with bone involvement under treatment with Ra^223^ alone develop visceral progression [103].

The most commonly measured serum marker, PSA, has obvious limitations, such as the possibility of radiological progression without elevation of this marker. New serum biomarkers, such as circulating tumor cells, have shown promising results but cannot be considered conclusive with poor correlations with the response as evaluated by CT or PET [27]. In this manner, new imaging modalities may contribute to the improved management of advanced prostate carcinoma by defining the presence and extent of tumors as well as the correct and early evaluation of response to treatment.

### Computed tomography

CT evaluates the soft tissue response following the RECIST v1.1 criteria and allows the determination of response, progression, or stability. However, it presents apparent limitations in the evaluation of the response of bone disease; for example, the appearance of new sclerotic lesions cannot be considered, per se, a criterion of progression [32]. Both CT and scintigraphy present difficulties in assessing the heterogeneity of response to treatment of local and distant disease [104].

### Diffusion-weighted WB-MRI

The advantages of the use of diffusion-weighted WB-MRI in the therapeutic monitoring of metastatic bone disease have already been discussed in the previous sections. The use of diffusion-weighted WB-MRI is not yet included in the assessment of high-risk PCa by the European Association of Urology (UAE) guidelines; however, the European Organization for Cancer Research and Treatment (EORTC) does recommend its inclusion in the diagnostic algorithm of PCa to identify oligometastatic disease and assess the response to treatment of metastases [105]. Furthermore, the Metastasis Reporting and Data System for Prostate Cancer (MET-RADS-P) guidelines have been published to improve reproducibility in the acquisition, comparison and reading of WB-MRI for the evaluation of metastatic PCa [106]. In CRPC patients, the use of this staging system has confirmed that the extent of bone metastases and the presence of visceral lesions are associated with shorter cancer-specific survival. These initial results open the door to using the MET-RADS-P score as a prognostic imaging biomarker for CRPC [107].

This technique is not affected by the “flare” effect of bone scintigraphy, allowing a quantitative and more reliable determination of the metastatic tumor load, which has prognostic implications and minimizes the use of ineffective therapies in patients with a low tumor load. Another field of application is the improvement in the detection of oligometastatic disease, allowing local rather than systemic treatments in this group of patients. Especially relevant is the more accurate and early detection of changes induced by therapy, introducing the possibility of differentiating between response, progression and stability, which improves the stratification of patients and therapeutic selection and, expectedly, final patient outcomes [32]. In this sense, quantitative biomarkers derived from diffusion-weighted sequences, such as ADC measures and tumor volume measured by means of DWI, correlate with serum biomarkers and clinical response to olaparib (inhibitor of the poly ADP-ribose polymerase enzyme) in patients with advanced PCa [108]. Another field of development for diffusion-weighted WB-MRI is the evaluation of tumor heterogeneity, an important factor in the response to therapy and in the development of resistance to treatment. Finally, this technique opens the possibility of conducting imaging biopsies to improve the understanding of the mechanisms of resistance to treatment by categorizing the genetic and molecular profiles of metastatic PCa and improving therapeutic selection [27].

### Bone scintigraphy

BS with ^99m^Tc-HDP is the most commonly used method to evaluate metastatic bone disease in patients with PCa as part of the validated criteria of the Prostate Cancer Working Group 3 to detect progression after treatment [109]. A meta-analysis with 1102 patients studied using planar BS and SPECT yielded combined sensitivity and specificity values to detect bone metastases of 79 and 82%, respectively [43]. The scintigraphic progression criterion requires the identification of at least two new bone lesions in consecutive studies. If detected, new lesions should be confirmed in a subsequent BS performed after at least 6 weeks to exclude the possible “flare” effect [109].

### Positron emission tomography

The usefulness of ^18^F-FDG-PET in prostate cancer is directly related to the phase of the disease. However, globally, it has a low yield due to poor avidity for glucose in prostate tumor cells, and the low uptake in metastatic lesions tends to decrease with androgen deprivation therapy or chemotherapy. However, tumors with more aggressive tumor biology than a Gleason score > 7 (clinically significant PCa) tend to have a high glycolytic rate [110].

In any case, PET with choline analogs has shown a higher rate of identification of tumor recurrence (58%) in both prostatic, lymphatic and bone bed lesions with an overall sensitivity and specificity of 86 and 93%, respectively [111].

Regarding the assessment of response to treatment, ^18^F, ^11^C-choline, ^68^ Ga or ^18^ F-PSMA offer the advantage of simultaneously evaluating the response of soft tissue and bone involvement, detecting signs of progression before CT and BS and obtaining a correlation between the decrease in SUV and overall survival.

In a recent study conducted in 177 patients with prostate cancer and 443 metastatic bone lesions and aimed at assessing the response of bone metastases by CT and PSMA-PET volumetric parameters: whole-body total-lesion PSMA, whole-body PSMA-tumor volume, as well as the established maximum standard uptake values (SUVmax) and mean standard uptake values (SUVmean), an association was determined between SUVmean, Gleason Scores, lesion classification, and serum-PSA levels but not for CT-derived bone density measurements before and after therapy (*p* > 0.05). Additionally, a highly significant correlation was observed for changes in PSMA-tumor volume, whole-body total-lesion PSMA, and serum PSA levels (*p* < 0.001) [112].

## Perspectives for the future

Currently, imaging techniques can provide quantitative information on the structural, functional and molecular aspects of cancer. Multiple quantitative biomarkers are derived from their processing, which allows studying new aspects of tumor biology, such as heterogeneity. The set of multivariate analysis techniques for these imaging biomarkers to detect new diagnostic, prognostic or predictive information on the response to treatment is called radiomics and has begun to be used in the evaluation of bone metastases. Thus, radiomics allows the successful differentiation between metastatic and nonmetastatic vertebral lesions in oncological patients investigated with MRI [113] and, more interestingly, allows prediction of the risk of metastatic bone disease in PCa using MRI texture analysis of the primary tumor [114].

Both in the therapeutic spectrum and in what concerns us now, namely, diagnosis, the development of nuclear medicine is fundamentally aimed at the synthesis of new radiopharmaceuticals with radiotherapeutic capacity and with greater and more selective affinity to the tumoral tissue under study, all to apply a personalized methodology to each histological phenotype.

In addition, the new diagnostic focus has been combined in recent years with the use of hybrid SPECT/CT, PET/CT and, more recently, PET/MRI. In addition to offering the possibility of integrating the functional/molecular information of PET in a single acquisition with the high-resolution anatomical information of MRI together with the functional information proportionated by DWI, this last modality could provide new and unique information additional to that obtained through the different PET radiotracers [115].

The radiopharmaceutical used in most PET/MRI studies is ^18^F-FDG, showing a greater SE in a group of 109 patients than that for ^18^F-FDG-PET/CT in the detection of bone metastasis in breast cancer (96 and 85%, respectively) with an SP of 99% [116, 117]. In addition, PET/MRI showed a greater number of bone metastases than PET/CT in 67 cancer patients with different tumor cell lines [115]. However, the SE of the technique is associated with the radiopharmaceutical used; therefore, new advances are directed to the application of new PET/MRI radiopharmaceuticals with favorable pharmacokinetic characteristics. Additionally, the use of ^18^FDG PET-MRI for staging breast cancer patients at high risk for metastases results in a treatment regimen in 14% of patients compared to a traditional staging imaging algorithm (including X-ray mammography, breast ultrasonography, chest plain radiography, bone scintigraphy, and ultrasonography of the liver and axillary fossa) [118].

Another study has shown a moderate but significant inverse correlation between increased choline metabolism and ADC values of bone metastases from prostate cancer using ^8^F-choline-PET/MRI [119]. In addition, Na[^18^F]F/[^18^F]FDG-PET/MRI permits the identification of more skeletal lesions than bone scintigraphy and the additional identification of extraskeletal disease in breast and prostate cancer patients [120].

^68^Ga-PSMA-PET/MRI scans show a high detection rate of prostate cancer recurrence even at very low PSA levels (< 0.5 ng/mL). In addition, at these levels, extrapelvic disease can be detected in 25% of cases [121]. In addition, recent data support that limited pelvic PSMA-PET/MR is equivalent to PET/CT in the assessment of metastatic regional lymph nodes and bone lesions and is superior to PET/CT with regard to capsular invasion and seminal vesicle involvement [122].

Despite these promising results, PET/MRI continues to be a technique with low accessibility due to its cost and the limited availability of hybrid equipment.

## Conclusions

Unfortunately, the metastatic bone disease remains a common clinical problem, particularly in patients with breast and prostate cancers. Classical imaging techniques, such as CT and bone scanning, have shown limited sensitivity in the detection and, more importantly, in the response assessment of these entities. Recent results support the use of next-generation imaging techniques, including whole-body diffusion-weighted MRI, PET/CT and PET/MRI with novel radiopharmaceuticals instead of the classic combination of CT and bone scans, due to improved results in detection, staging and categorization of the response to local and systemic therapies. In a practical example of precision and personalized medicine, the use of next-generation imaging technologies in patients with metastatic bone disease combined with information from other genetic and molecular diagnostic techniques, which offer complementary capacities, offers clinicians a wide armamentarium of diagnostic, predictive and prognostic biomarkers to improve patient management and outcome.

## Data Availability

Not applicable.
